# SAPHO syndrome complicated by IgG4-related ophthalmic disease: a case report and literature review

**DOI:** 10.3389/fimmu.2025.1563542

**Published:** 2025-04-24

**Authors:** Chenxiao Liu, Tingting Chen, Yanyan Wang, Qi Wang, Hao Hu, Huanhuan Chen

**Affiliations:** ^1^ Department of Endocrinology, The Affiliated Suzhou Hospital of Nanjing Medical University, Suzhou Municipal Hospital, Gusu School, Nanjing Medical University, Suzhou, Jiangsu, China; ^2^ Department of Endocrinology, The First Affiliated Hospital of Nanjing Medical University, Nanjing, Jiangsu, China; ^3^ Department of Endocrinology, Nanjing Meishan Hospital, Nanjing, Jiangsu, China; ^4^ Department of Rheumatology and Immunology, The First Affiliated Hospital of Nanjing Medical University, Nanjing, Jiangsu, China; ^5^ Department of Radiology, The First Affiliated Hospital of Nanjing Medical University, Nanjing, Jiangsu, China

**Keywords:** SAPHO syndrome, IgG4, ophthalmic disease, TNF-a (tumor necrosis factor a), immune disorders

## Abstract

**Introduction:**

Synovitis, acne, pustulosis, hyperostosis, and osteitis (SAPHO) syndrome is an extremely rare condition with nonspecific clinical signs and symptoms.

**Case description:**

Here, we present the case of a 54-year-old Chinese woman with an 8-year history of recurrent furuncles and a 6-year history of clavicular pain. Initially, computed tomography (CT) showed nonspecific changes. The patient was treated with nonsteroidal anti-inflammatory drugs for symptomatic relief; however, clavicular symptoms recurred intermittently. Two years before the current presentation, the patient experienced ocular discomfort from bilateral lacrimal gland enlargement and extraocular muscle thickening. She exhibited elevated IgG4 levels and was diagnosed with IgG4-related ophthalmic disease. Treatment with low-dose glucocorticoids slightly improved her clavicular symptoms. However, over the past month, her clavicular pain worsened. Recent CT and magnetic resonance imaging revealed a deformity of the right clavicular cortex near the end, with poor bone quality and continuity, and bone scintigraphy revealed intense radiotracer uptake in the sternoclavicular region. Consequently, the patient was diagnosed with SAPHO syndrome.

**Conclusion:**

While clinical manifestations and imaging are helpful in narrowing the differential diagnosis, biopsy and histopathological examinations are necessary to confirm SAPHO syndrome. Regulation of TNF-α may be a therapeutic option for bone pain in this patient. In patients with an initial presentation of abnormal IgG4 levels, physicians must maintain a high index of suspicion to ensure appropriate treatment.

## 1 Introduction

SAPHO syndrome (synovitis, acne, pustulosis, hyperostosis, and osteitis) is an exceptionally rare condition characterized by a chronic inflammatory response affecting both the dermatological and osteoarticular systems (1, 2). Although its etiology remains unknown, SAPHO syndrome is widely considered an autoinflammatory disorder that may be triggered by immunological dysregulation, microbial infections, and genetic susceptibility.

Oculopathy is rarely accompanied by SAPHO syndrome. In this report, we describe an unusual case exhibiting elevated IgG4 concentrations and ocular disease involving thickened extraocular muscles, retroocular inflammatory exudation, and significant lacrimal gland enlargement.

## 2 Case description

A 54-year-old woman with an 8-year history of recurrent palmoplantar pustulosis, a 6-year history of sternoclavicular joint pain, and a 2-year history of eye discomfort and swelling was admitted to the rheumatology department of our hospital in July 2024.

Eight years before the current presentation, the patient was diagnosed at a nearby outpatient clinic with palmoplantar pustulosis, presenting as skin ulcers and erosions on both feet and the fingers; isotretinoin was prescribed for this condition. Six years prior, she experienced recurrent pain in the right clavicular area. Computed tomography (CT) revealed mild nonspecific changes in the clavicle. She had since intermittently taken nonsteroidal anti-inflammatory drugs (NSAIDs) to alleviate her bone pain.

**Figure 1:**
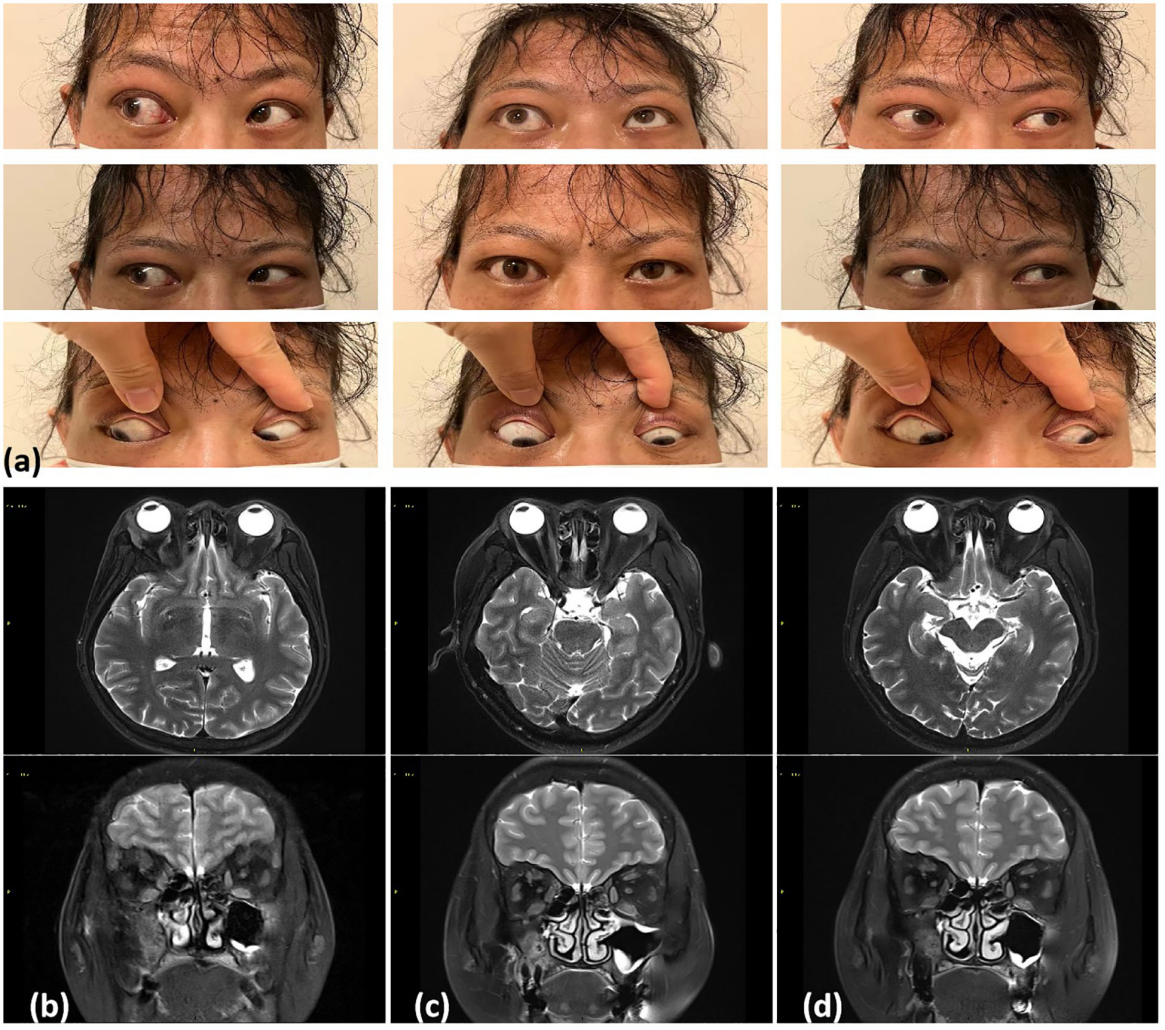
**(a)** Two years ago (November 2022), physical examination revealed limited outward rotation of the right eye and mild limitation in the downward rotation of both eyes. SE T1 weighted MR axial images of the orbits with fat saturation postgadolinium. **(b)** November 2022: Bilateral lacrimal gland enlargement with slight thickening and swelling of the inferior rectus muscles on both sides and the right medial rectus muscle. **(c)** March 2023: Lacrimal gland enlargement and significant improvement in the thickening of the eye muscles. **(d)** September 2023: Near-complete normalization of changes in the eye muscles.

Two years prior, the patient experienced exophthalmos, photophobia, and lacrimation, particularly in her right eye. Laboratory evaluation revealed elevated serum IgG4 levels (3.16 g/L; reference range, <2 g/L). Intraocular pressures were 19.7 and 18.4 mmHg for the right and left eyes, respectively (**Figure 1a**). Magnetic resonance imaging (MRI) revealed right eyeball protrusion with exophthalmos up to 21.3 mm, accompanied by bilateral lacrimal gland enlargement and slight thickening and swelling of the inferior rectus muscles and right medial rectus muscle (**Figure 1b**). Another remarkable comorbid condition was thyroid dysfunction, which was TRAb-positive (8.4 IU/L; reference range, 0–1.75). After treatment with thiamazole 10 mg tablets twice daily for approximately 20 days, hypothyroidism developed and has been well compensated with levothyroxine (current dose, 25 μg/day). Considering these findings and after discussing the risks, she was prescribed prednisone 20 mg once daily. The dose was gradually tapered and discontinued 6 months prior. During the treatment, her photophobia, tearing, and eye discomfort gradually improved. A follow-up MRI confirmed improvements to the enlarged lacrimal glands and thickened extraocular muscles (**Figures 1b, c**), with a decrease in the degree of eyeball protrusion (**Figure 1d**). However, her sternoclavicular symptoms (pain, severe swelling, tenderness, and inflammation) began to progress 1 month prior, causing difficulties turning her body and lifting the right shoulder. She denied recent infections, medication, or stress. She had no family history of SAPHO syndrome or spondyloarthropathy.

**Figure 2:**
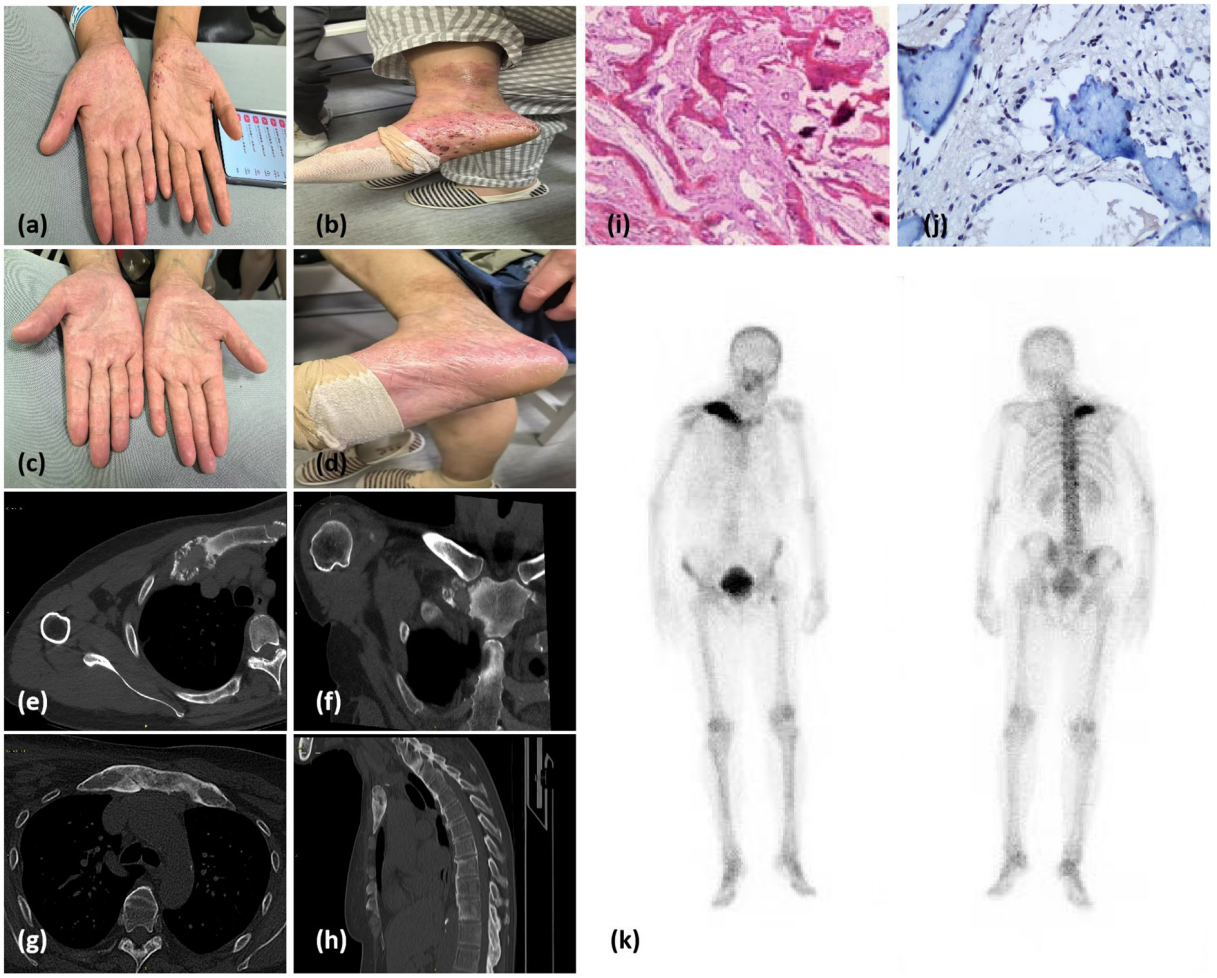
**(a, b)** Pustules on the palms and feet suggestive of palmoplantar pustulosis. **(c, d)** Resolution of palmoplantar pustules after treatment. **(e, f)** Chest and shoulder computed tomography (CT) scans from 2018 showing mild, nonspecific bone destruction and sclerosis on the surface of the right sternoclavicular joint. **(g, h)** CT images of the anterior chest wall and right clavicle from 2024 showing multiple foci of bone marrow edema and discontinuous cortical bone. **(i)** Histology of the clavicle bone. Hematoxylin and eosin staining showing woven bone hyperplasia, focal fibrous tissue proliferation, and mild chronic inflammatory infiltration. **(j)** Immunohistochemical of the clavicle bone. The image more clearly demonstrates TNF-α aggregation in osteoblasts and lymphocytes within bone tissue. **(k)** Whole-body bone scintigraphy image showing intense radiotracer uptake in the T10 and T12 vertebrae, the top of the left femur, and the sternoclavicular region.

During the patient’s most recent visit to our hospital, she was 156.0 cm tall, weighed 60 kg, and had a blood pressure of 135/88 mmHg. Physical examination revealed slight swelling of both eyelids with unrestricted eye movement, no enlargement or tenderness of the thyroid gland, the presence of pustular eruptions on both hands and feet (**Figures 2a, b**), and swelling and tenderness of the right sternoclavicular joint.

Blood tests indicated that total cholesterol, liver function markers, and urea/creatinine levels were within normal ranges. Thyroid function tests indicated mild hyperthyroidism. Her visual acuity was 0.8 in the right eye and 1.0 in the left eye. Pupil reactions, color vision, and fundoscopic findings were normal in both eyes.

Six years prior, chest and shoulder CT scans revealed mild, nonspecific bone destruction and sclerosis on the surface of the right sternoclavicular joint (**Figures 2e, f**). Recent CT and MR images revealed cortical bone deformation at the proximal end of the right clavicle, with poor bone-mass continuity. Multiple foci of bone marrow edema were also present in the right clavicle, the first costosternal joints, and the sternal manubrium (**Figures 2g, h**). A bone biopsy was performed, and histopathological findings were consistent with chronic osteomyelitis, with no evidence of infection or tumors (**Figure 2i**). Immunohistochemical analysis of bone tissue revealed focal accumulation of TNF-α surrounding smooth muscle cells (**Figure 2j**). Bone scintigraphy using 99mTc–methylene diphosphonate showed intense radiotracer uptake at the T10 and T12 vertebrae, the proximal left femur, and the sternoclavicular region (**Figure 2k**). Following treatment with NSAIDs (*celecoxib* 200 mg daily) and *Tripterygium wilfordii polyglycoside* (TwHF) (0.5 g three times daily), the patient’s bone pain resolved, and the skin lesions subsided (**Figures 2c, d**).

## 3 Discussion

The clinical presentation of SAPHO syndrome necessitates careful differentiation from several inflammatory and infectious conditions. Key differential diagnoses include chronic recurrent multifocal osteomyelitis, psoriatic arthritis, ankylosing spondylitis, and infectious osteomyelitis. This patient’s combination of palmoplantar pustulosis, sternoclavicular hyperostosis, and IgG4-ROD-like ocular involvement favored SAPHO syndrome over isolated autoimmune or infectious etiologies.

Accumulating evidence redefines SAPHO syndrome as a multisystem inflammatory disorder characterized by heterogeneous extrapulmonary manifestations extending beyond its hallmark osteoarticular-cutaneous triad. A 2023 meta-analysis of 487 confirmed SAPHO cases (PRISMA-compliant) demonstrated extracutaneous organ involvement in 41% (95% CI 36–47%), with distinct patterns: gastrointestinal (12%): ileocecal Crohn’s disease predominance (8.2%, *P*<0.01 vs the general population) with concurrent anterior chest wall osteitis; pulmonary (6.3%): non-granulomatous interstitial lung disease (HRCT pattern: 73% ground-glass opacities) and costosternal pleuritis; neurological (4.7%): cranial neuropathies secondary to skull base hyperostosis (MRI-detected foraminal stenosis in 82% of symptomatic cases) (3). Recent case series documenting extra-cutaneous involvement, including inflammatory arthritis (4), sternoclavicular hyperostosis (5), and rare visceral manifestations such as glomerulonephritis (6). Population-based studies highlighting the prevalence of constitutional symptoms (e.g., fever, fatigue) in 30-40% of SAPHO patients (2). Emerging evidence of autoimmune overlap, including associations with uveitis and inflammatory bowel disease (7).

Orbital and adnexal involvement in IgG4-related diseases presents diverse characteristics depending on the location of lymphoplasmacytic infiltration. Sclerosing dacryoadenitis, trigeminal nerve enlargement, and orbital fat infiltration are characteristic of IgG4-ROD. Less frequently, IgG4-ROD can affect the lacrimal drainage system, sclera, and conjunctiva (8). An elevated serum IgG4 level is neither necessary nor specific to the disease, despite its inclusion in most of the diagnostic criteria for IgG4-ROD (9).

Our patient had a history of thyroid dysfunction, with TRAb values rapidly becoming negative. Orbital MRI indicated significantly enlarged lacrimal glands and signs of eye muscle involvement. Although the patient’s serum IgG4 level was mildly elevated, her ocular lesions were consistent with those of IgG4-ROD. These findings align with the first and third criteria established in 2015 (10) for a possible IgG4-ROD diagnosis. Regrettably, further pathological examinations and measurements of IgG4+ cell levels in the ocular lesions were not conducted.

The immunologic interface between SAPHO and IgG4-related disease merits particular attention. In this specific case, a disrupted immune response may have participated in triggering the symptoms, especially considering the patient’s history of elevated serum IgG4 levels and long-term thyroid dysfunction. Elevated serum IgG4 levels have also been reported in other patients with SAPHO syndrome (11). The proliferation of circulating CD4+ cytotoxic T lymphocytes may elevate the helper T (Th)1 cell count in the peripheral blood of patients with IgG4-related disease (12). Th1 chemokines and their related receptors play key roles in the pathogenesis of thyroid-associated ophthalmopathy, particularly during the active phase. Notably, patients with immunological dysfunction often exhibit abnormalities in tumor necrosis factor-alpha (TNF-α)-producing Th1 cells. Bone and cartilage biopsy specimens from patients with SAPHO syndrome exhibit increased TNF-α production and expression.

Patients with SAPHO syndrome exhibit a higher prevalence of autoimmune thyroid conditions (13). These individuals express normal thyroid-specific genes across various skin cell types (14). Additionally, the thyroid expresses TNF-α receptors, which enables cytokines to directly influence thyroid function (e.g., in antigen presentation and hormone production) and exacerbate autoimmune thyroid disorders (15). Consequently, TNF-α has been proposed as a therapeutic target for autoimmune thyroid disease linked to Th17/Th1 imbalance (16). In support of autoimmune pathogenesis, individuals with both SAPHO syndrome and chronic recurrent multifocal osteomyelitis exhibit elevated TNF-α levels in the bloodstream (17), suggesting that excessive TNF-α could disrupt neutrophil behavior, inciting a feedback loop that intensifies the inflammatory response through the activation of keratinocytes (18).

TNF-α dysregulation and the associated changes to thyroid hormone can cause disturbances similar to those observed in euthyroid sick syndrome (19), TNF-α levels are elevated in hypothyroidism regardless of etiology or phase, and TNF-α administration has occasionally converted hyperthyroidism to hypothyroidism (20, 21). Mechanistic studies propose TNF-α-mediated thyroid peroxidase antibody production, creating a self-perpetuating inflammatory cycle. This aligns with our patient’s thyroid dysfunction trajectory, where methimazole-induced euthyroid state coincided with cutaneous and osteoarticular flare-ups.

In our study histopathological evaluation demonstrated substantial TNF-α production in the lesional microenvironment. This observation aligns with independent investigations revealing an increase in TNF-α+ macrophage density surrounding osteoclasts in SAPHO syndrome compared to osteoarthritis controls. These findings collectively suggest mechanistic parallels with IgG4-related disorders, wherein TNF-α orchestrates fibroblast-to-myofibroblast transition through NF-κB-mediated upregulation of fibrogenic mediators, ultimately driving progressive tissue fibrosis (22). This cytokine synergy may explain the therapeutic efficacy of TNF inhibitors observed in refractory SAPHO cases. Clinical studies have shown that TwHF significantly reduces joint tenderness and swelling in patients, while also alleviating inflammatory responses and improving immune function (23). Animal experiments have demonstrated that TwHF can decrease the levels of the inflammatory factor TNF-α in rats with collagen-induced arthritis, thereby ameliorating joint inflammation and reducing pathological damage to the joints (24). A randomized controlled trial demonstrated the rapid onset of action of TwHF in treating SAPHO syndrome, achieving maximum efficacy within the first 2–4 weeks and a stable level of effectiveness maintained throughout the study period. Additionally, TwHF demonstrated significant efficacy and reliable safety (25, 26). Thus, we treated our patient with TwHF (150 mg/d). Within 4 weeks, the patient experienced relief from palmoplantar pustules (**Figures 2c, d**) and sternal pain, without any discomfort in the eyes. Celecoxib was gradually tapered, and the patient continued TwHF treatment. At the most recent follow-up, all symptoms had resolved, and the patient remains under treatment.

In conclusion, our case reinforces SAPHO syndrome’s designation as a multisystem inflammatory disorder rather than a purely osteoarticular entity. The identification of ocular involvement through IgG4-ROD underscores the need for comprehensive organ system evaluation. We propose a diagnostic algorithm incorporating the following:

1. Routine ophthalmological evaluation in SAPHO patients, including orbital MRI for lacrimal gland assessment;
2. Serum IgG4 quantification and TNF-α profiling at diagnosis to guide targeted therapy.

Further characterization of the SAPHO-IgG4 spectrum through international registries will be crucial for optimizing therapeutic paradigms.

## Data Availability

The raw data supporting the conclusions of this article will be made available by the authors, without undue reservation.
